# Reply: Study design and statistics in epidemiology of breast cancer

**DOI:** 10.1038/sj.bjc.6603371

**Published:** 2006-10-03

**Authors:** M Garcia-Closas, L A Brinton, W F Anderson, M E Sherman

**Affiliations:** 1 Division of Cancer Epidemiology and Genetics, National Cancer Institute, NIH 6120 Executive Boulevard Executive Plaza South, Room 7076, MSC 7234, Rockville, MD 20852-7234, USA

**Sir**,

We thank Drs Maraqa and Lansdown for providing us with an opportunity to clarify concerns about the generalisability and validity of the findings in our recently published article on heterogeneity of breast cancer risk factors by tumour characteristics in a population-based study conducted in Poland (Garcia-Closas *et al*, 2006). We are confident that the fundamental conclusions of our study are valid and can be generalised to most other Western populations for the reasons outlined below.

The authors note that the percentage of oestrogen receptor (ER)-negative tumours in our study population was higher than in most other Western populations. The percentage of ER-negative tumours in the Polish population for all tumour types combined was 34% (the figure of 41% quoted by Maraqa and Lansdown was for ductal NOS tumours only), compared to 23% reported in the Surveillance, Epidemiology and End Results (SEER) in the US for the years of 2000–2002 and a similar age range as in our report (Surveillance, 2005). There are several possible explanations for this apparent difference. First, the relatively low percentage of women having screening mammography in Poland compared to other Western populations, such as the UK or US, probably led to greater underdetection of slow-growing indolent ER-positive tumours compared with ER-negative tumours. Second, criteria for distinguishing weakly ER-positive from ER-negative tumours vary, and in countries like the US, there is a tendency to favour characterisation of weakly positive tumours as ER positive, to provide more women options for tamoxifen treatment (Harvey *et al*, 1999). Finally, exposures that might increase the risk of ER-positive tumours, such as high body mass index among postmenopausal women (Althuis *et al*, 2004), were less common in the Polish population than in other countries such as the US. Respectively, the percentage of women who were overweight (⩾25 kg/m^2^) among white non-Hispanics in the US (Flegal *et al*, 2002) and Polish study subjects were 49 *vs* 22% for 20–39 year olds, 61 *vs* 36% for 40–59 year olds and 66 *vs* 40% among those 60 or older.

The primary goal of our report was to assess the modification of the associations between aetiologic exposures and breast cancer risk by tumour subtypes, using a novel statistical approach to account for correlated tumour characteristics, including hormone receptor status. Our results were generally consistent with previous studies, thus providing support for both the validity and generalisability of our conclusions. Although our estimates of overall relative risk for exposures that are modified by ER status are valid, we recognise that they may differ from those found in populations with a lower percentage of ER-negative tumours. It was notable that the direction and magnitude of overall associations of most aetiologic exposures and breast cancer risk in Poland were similar to previously published reports, with the exception of obesity among postmenopausal women, which has been linked to greater risk for ER-positive as compared to ER-negative tumours in many studies (Althuis *et al*, 2004).

Our analyses also utilised standard statistical approaches to adjust for correlated risk factors. Although residual confounding is always possible, the contention that associations may have been biased by a failure to account for factors correlated with mammographic screening (e.g., education, HRT use) would not apply. As shown in Table 1 of our report, there were some differences in prior screening proportions between the cases and controls (62 *vs* 54%), which are likely to reflect increased reporting by cases of recent mammograms performed for breast cancer symptoms.

Finally, concerns were also expressed regarding potential error with histologic classifications in our study. The levels of agreement between Polish and US pathology results for the classification of ductal (80%) and lobular (68%) tumours were similar to what would be expected in comparable reviews in other settings, and the main disagreements were for the classification of mixed tumours (18%), largely attributable to differences in terminology. The US review was performed to afford maximal opportunities for exploring aetiologic relationships, whereas the Polish review was performed for clinical management. Accordingly, the US review applied stringent criteria for classifying tumours as pure ductal or lobular carcinomas, placing more tumours in the mixed category. Inclusion of cases without the US review could have diluted differences between ductal and lobular tumours, but should not have created differences had they not been present.
